# A Mendelian Randomization Study of the Effect of Tea Intake on Type 2 Diabetes

**DOI:** 10.3389/fgene.2022.835917

**Published:** 2022-03-29

**Authors:** Yanan Zhang, Ruiqing Wang, Xinhua Tang, Yanjun Wang, Ping Guo, Shukang Wang, Jing Liu

**Affiliations:** ^1^ Department of Biostatistics, School of Public Health, Cheeloo College of Medicine, Shandong University, Jinan, China; ^2^ Department of Hematology, Qilu Hospital of Shandong University, Jinan, China; ^3^ School of Cyberspace Security, Shandong University of Political Science and Law, Jinan, China

**Keywords:** type 2 diabetes, tea intake, causal association, genome-wide association study, Mendelian randomization

## Abstract

**Background:** The association reported between tea intake and type 2 diabetes (T2D) is inconsistent in previous studies and remains controversial. We aimed to explore the causal relationship between tea intake, T2D, and glycemic traits including hemoglobin A1c (HbA1c), fasting plasma glucose (FPG), fasting serum insulin (FSI), and homeostasis model of insulin resistance (HOMA-IR) levels.

**Methods:** A 2-sample Mendelian randomization (MR) was performed using summary statistics from large-scale genome-wide association studies of tea intake from the UK Biobank, T2D from the DIAGRAM consortium, and glycemic traits from the Magic consortium. The findings were verified through sensitivity analyses using various MR methods with different model assumptions and by comprehensively evaluating the influence of pleiotropy effects and outliers.

**Results:** With the use of a two-sample MR with inverse variance-weighted method, the odds ratio per unit SD change of tea intake (SD: 2.85 cups/day) for T2D, HbA1c, FPG, FSI, and HOMA-IR levels was 0.949 (95% CI 0.844–1.067, *p* = 0.383), 0.994 (95% CI 0.975–1.013, *p* = 0.554), 0.996 (95% CI 0.978–1.015, *p* = 0.703), 0.968 (95% CI 0.948–0.986, *p* = 0.001), and 0.953 (95% CI 0.900–1.009, *p* = 0.102), respectively. The results were consistent with those of the other six methods that we used with different model assumptions, suggesting that the findings were robust and convincing. We also performed various sensitivity analyses for outlier removal, pleiotropy detection, and leave-one-out analysis.

**Conclusion:** Our MR results did not support the causal effect of tea intake on T2D and crucial glycemic traits. These findings suggest that previous observational studies may have been confounded.

## Introduction

Diabetes has become a public health issue worldwide with increasing prevalence. It is estimated that the total number of patients with diabetes will exceed 550 million by 2030, and 90% of them will have type 2 diabetes (T2D) (Yamaji et al., 2004). T2D is a metabolic disorder resulting from both genetic and environmental factors and is characterized by insulin insensitivity, insulin deficiency, and impaired biological function (Li et al., 2020a). Lifestyle intervention for weight loss through energy restriction and physical activity was reported to be more effective than drug intervention in reducing the incidence of T2D (Knowler et al., 2002). In addition, dietary factors, including but not limited to the intake of tea, vegetables, fruits, and whole grains, can play a vital role in preventing the onset of T2D and may be useful in glycemic control (Iso et al., 2006; Mamluk et al., 2017; McRae, 2018; Qian et al., 2019). Among these, the relationship between tea intake and T2D was of particular interest to us because of the complexity of the composition of tea (Fukino et al., 2005; Kim et al., 2005; Babu et al., 2007; Babu et al., 2008; Brown et al., 2009; Ramadan et al., 2009; Brown et al., 2011; Suzuki et al., 2012; Wu et al., 2012; Yang and Hong, 2013; Meng et al., 2019) and the inconsistent results from previous studies about the effect of tea intake on T2D (van Dam and Hu, 2005; Boggs et al., 2010; Oba et al., 2010; Hayashino et al., 2011; van Woudenbergh et al., 2012; Bhupathiraju et al., 2013; Yang et al., 2014a; Yang et al., 2014b; Liu et al., 2018; Nie et al., 2021).

Tea contains various components such as polyphenols, caffeine, tannins, vitamins, and saponins (Kim et al., 2005; Suzuki et al., 2012; Yang and Hong, 2013). The bioactive components may play an important role in lowering the fasting plasma glucose (FPG) and improving insulin resistance and glucose metabolism (Meng et al., 2019). It has been reported that polyphenols, which include catechins, flavanols, theaflavins, and thearubigins, can reduce the risk of T2D as well as the associated complications (Ramadan et al., 2009). Although animal experiments (Babu et al., 2007; Babu et al., 2008) and human clinical studies (Wu et al., 2012) have shown that tea extracts can reduce FPG or hemoglobin A1c (HbA1c) levels and improve insulin resistance, many randomized controlled trials (RCTs) conducted with human participants have not confirmed this finding (Fukino et al., 2005; Brown et al., 2009; Brown et al., 2011). Therefore, investigating the causal relationship between tea intake and T2D would be significant in aiding the prevention of T2D and devising suitable interventions.

The association between tea intake and T2D has been well documented in observational epidemiological studies. However, the studies have not been conclusive. For example, some studies reported that tea drinking may reduce the risk of T2D (van Dam and Hu, 2005; van Woudenbergh et al., 2012; Bhupathiraju et al., 2013; Yang et al., 2014a; Yang et al., 2014b; Nie et al., 2021), while some reported that it may even increase the risk of T2D (Hayashino et al., 2011; Liu et al., 2018). Some studies failed to detect any association between T2D and tea drinking (Boggs et al., 2010; Oba et al., 2010). The inconsistent and unreliable results from traditional epidemiology studies may be partly due to the interference of reverse causality and the influence of various other possible confounding factors, particularly unobserved confounders. Inadequate adjustment for confounding factors can bias the association between tea intake and T2D. A better approach to assess the evidence on their causal relationship is required.

The Mendelian randomization (MR) has become a common statistical tool that uses single-nucleotide polymorphisms (SNPs) as genetic instrumental variables (IVs) to infer the causal relationship between exposure and outcome (Sleiman and Grant, 2010; Zeng and Zhou, 2019). It is generally believed that RCTs are the gold standard for investigating the causal relationship between exposure and outcome. However, RCTs have certain limitations, such as difficulty in implementation and associated ethical issues (Jones and Podolsky, 2015; Bothwell and Podolsky, 2016). MR is termed as a “natural” RCT (Bennett and Holmes, 2017) because the alleles that affect the exposure during the meiotic stage are assigned randomly. Using SNPs as genetic tool variables to infer the causal relationship between exposure and outcome can eliminate various confounding biases caused by known and unknown confounding factors. Further, possible reverse causality can be avoided by this method (Davey Smith, 2003). Moreover, owing to the relatively high measurement accuracy of SNPs, the measurement error will affect the MR results (Haycock et al., 2016). In addition, the large number of published genome-wide association studies (GWASs) provides a rich data resource (Swerdlow et al., 2016) for the implementation of the two-sample MR method. Collectively, it is possible to apply the MR method to study the causal relationship between variables such as lifestyle exposure and outcome or genetic factors and diseases (Yuan et al., 2020; Liu et al., 2021). Hence, we performed an MR study to explore the causal relationship between tea intake and T2D, which was followed by sensitivity analyses using various MR methods with different model assumptions.

## Materials and Methods

### Study Design

Therefore, we carried out a two-sample MR study using GWAS summary statistics with tea intake and T2D from two separate GWASs (Lawlor, 2016), followed by sensitivity analyses using various MR methods with different model assumptions. In addition, we explored the causal effects of tea intake on glycemic traits, which included HbA1c, FPG, fasting serum insulin (FSI), and homeostasis model of insulin resistance (HOMA-IR) levels. The complete details are provided in Supplementary Figure S1.

### Data Source

The two-sample MR method requires that the SNPs related to the exposure or outcome are derived from different studies that are based on a different sample set of the same ethnicity (Burgess et al., 2013). We used the tea intake GWAS from UK Biobank (UKBB) (data field 1488). Specifically, the UKBB is a large-scale cohort study comprising of participants aged 40–69 years from the United Kingdom (Bycroft et al., 2018). All enrolled participants at the baseline assessment were asked 29 questions regarding their diet using a touchscreen food-frequency questionnaire. To investigate tea intake, the participants of the UKBB study were asked how many cups of tea they drank each day, including black and green tea. Participants who responded <0 or >99 were excluded, and those who responded with >20 were asked to reconfirm. Finally, the reported range of tea intake was from 0 to 99 cups/day (mean and SD: 3.51 ± 2.85 cups/day). The tea intake genetic association data were obtained from the UKBB release 2 data (https://biobank.ctsu.ox.ac.uk/crystal/field.cgi?id=1488), in the Atlas of GWAS Summary Statistics (Watanabe et al., 2019).

The DIAGRAM consortium is a group of researchers who explore the underlying genetic architecture of T2D in large-scale studies. GWAS summary statistics on T2D in this study were obtained from the largest GWAS to date, which consisted of 32 sub-studies in the DIAGRAM consortium (http://diagram-consortium.org/) (Mahajan et al., 2018). Specifically, the tea intake GWAS with 10,570,778 SNPs for 373,481 individuals and the body mass index (BMI)-unadjusted T2D GWAS with 7,368,848 SNPs on 898,130 individuals with 74,124 cases and 824,006 controls were used. GWAS summary statistics on glycemic traits, which included HbA1c, FPG, FSI, and HOMA-IR levels, for this study were obtained from the Magic consortium (https://magicinvestigators.org/) (Manning et al., 2012; Chen et al., 2021).

### Selection of Instrumental Variables

**FIGURE 1:**
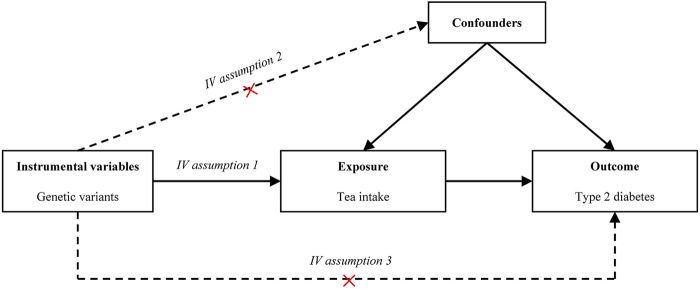
Causal diagram. The causal diagram for Mendelian randomization (MR) involves instrumental variables (IVs) and requires three assumptions: **(1)** Relevance. The IVs associate robustly with the exposure. **(2)** Independence. The IVs are not associated with observed or unobserved confounding factors for the exposure and outcome. **(3)** Exclusion restriction. The IVs only affect the outcome through exposure. Dotted lines represent a possible condition that the assumptions may be violated. Abbreviation: IV, instrumental variable.

MR has become a common statistical tool in observational studies to investigate the causal relationship between exposure variables (i.e., tea intake) and outcome variables (i.e., T2D). Standard MR analysis requires that the following three model assumptions must be satisfied (Lawlor et al., 2008) (Figure 1): 1) IVs are associated with the exposure, 2) IVs are not associated with observed or unobserved confounding factors, and 3) IVs only affect the outcome through the exposure. The first assumption can be directly tested using the observed data. However, the last two assumptions are often difficult to validate in practice. In the present study, we verified our findings using various MR methods under different model assumptions.

**FIGURE 2:**
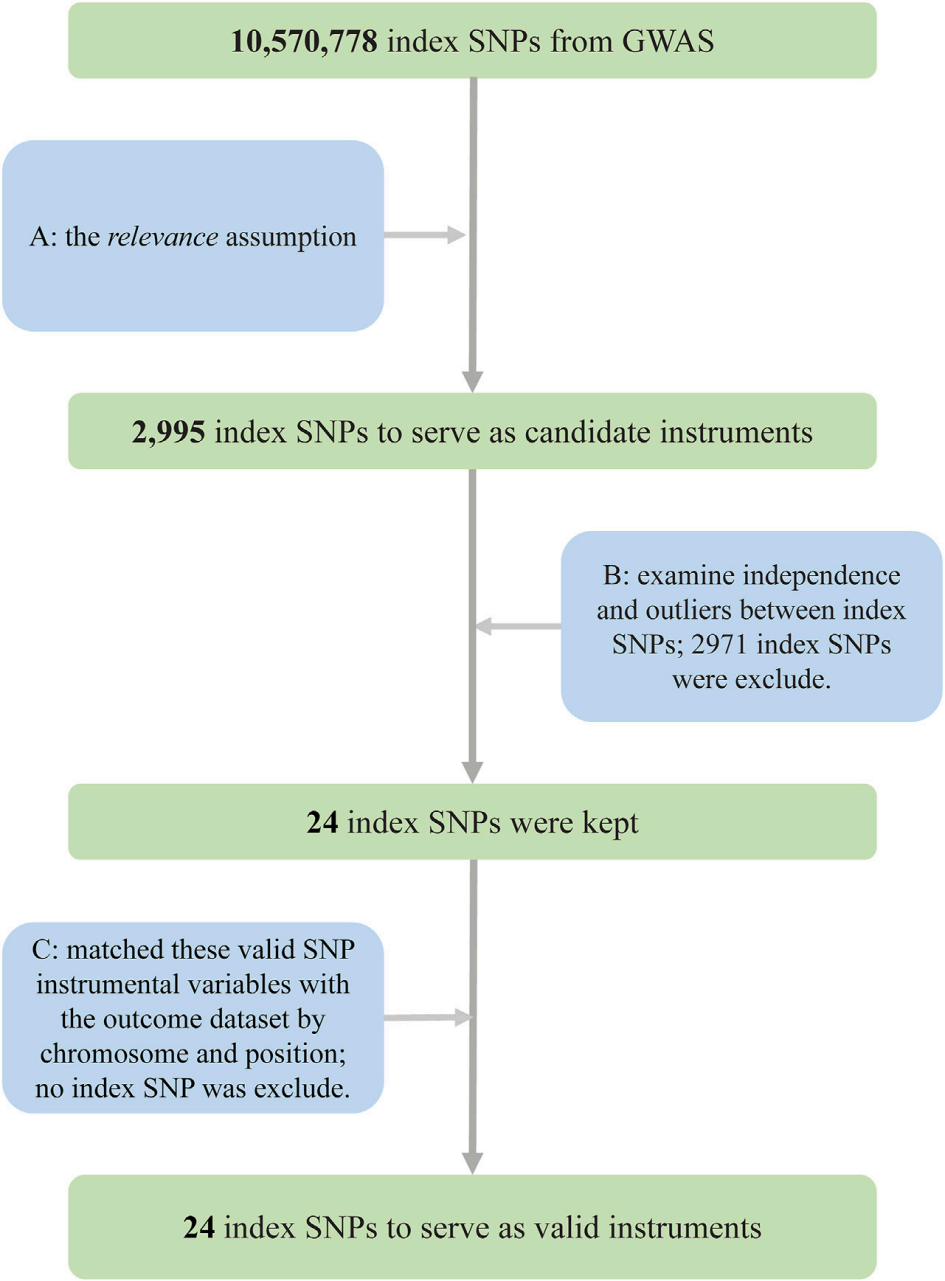
Flowchart for the selection of instrumental variables to investigate the causal effect of tea intake on T2D. **(A)** Selected SNPs significantly associated with the exposure (*p* = 5 × 10^−8^, at genome-wide threshold). **(B)** Obtained independent SNPs and examined the effect size outliers with linkage disequilibrium (LD) with *r* ^2^ < 0.01 or the physical distance more than 10,000 kb. **(C)** Harmonized candidate SNPs with the outcome dataset by chromosome and position Abbreviations: GWAS, genome-wide association study; SNPs, single-nucleotide polymorphisms; T2D, type 2 diabetes.

In our two-sample MR, IVs were selected through the following rigorous procedures (Figure 2): 1) we selected SNPs that were significantly associated with the exposure (*p* = 5 × 10^−8^, at genome-wide threshold); 2) we obtained independent SNPs and examined the effect size outliers with linkage disequilibrium (LD) with *r* ^2^ < 0.01 or a physical distance greater 10,000 kb, where *r* ^2^ is a measure of LD, a non-random association of alleles at two loci (Canzoniero and Rosenberg, 2008). We applied the clumping procedure of PLINK (version 1.90) (Purcell et al., 2007) using a reference dataset of the European population from the 1000 Genomes Project (Robinson et al., 2020), and 3) we harmonized candidate SNPs with the outcome dataset by chromosome and position. To evaluate weak instrumental bias, we calculated the *F* statistic, also known as the Cragg–Donald statistic (Burgess et al., 2017).

### Main Analysis

We applied a two-sample MR to investigate the causal effect between tea intake and T2D or glycemic traits using the random inverse variance-weighted (IVW) method (Burgess et al., 2013) as the main analysis. We assumed that the effect size and the variance for IV G_j_ on the continuous exposure X were ${\hat{\beta }}_{j}^{x}$ and var ( ${\hat{\beta }}_{j}^{x}$ ) (*j* = 1, 2, …, k), respectively, and we assumed that the effect size and the variance for the same IV were G_j_ on Y was ${\hat{\beta }}_{j}^{y}$ and var ( ${\hat{\beta }}_{j}^{y}$ ), respectively. The causal effect can be represented as Eq. 1.

$${\hat{\theta }}_{ivw}=\frac{\sum_{j=1}^{k}\text{var}{\left({\hat{\beta }}_{j}^{y}\right)}^{-1}{\hat{\beta }}_{j}^{y}{\hat{\beta }}_{j}^{x}}{\sum_{j=1}^{k}\text{var}{\left({\hat{\beta }}_{j}^{y}\right)}^{-1}{\left({\hat{\beta }}_{j}^{x}\right)}^{2}}\;$$ (1)

### Sensitivity Analyses

The results from the main analysis were validated by several different MR methods with different model assumptions: 1) MR-Egger regression, which is used to evaluate the directional pleiotropy of IVs according to their intercept (i.e., intercept *p* < 0.05) (Burgess and Thompson, 2017); 2) the weighted median method, which can provide consistent estimates when at least half of the instruments used in the analysis are valid (Bowden et al., 2016); 3) MR Mode-based estimate (MR-MBE) (Hartwig et al., 2017), which relaxes the strict IV assumptions; 4) the IVW method using robust regression (MR-Robust) (Slob and Burgess, 2020), which reduces the standard error of the estimates; 5) MR Robust Adjusted Profile Score (MR-RAPS) (Zhao et al., 2020), which is not influenced by both systematic and idiosyncratic pleiotropy; and 6) leave-one-out (LOO) cross-validation analysis (Noyce et al., 2017) and MR Pleiotropy RESidual Sum and Outlier (MR-PRESSO) analysis (Verbanck et al., 2018), which used outlier detection. In addition, we depicted diagnostic plots (e.g., SNP scatter plots and funnel plots) to illustrate the MR results. Supplementary Table S1 shows the comparison and complement of the different methods. In particular, funnel plots can visually detect the directional pleiotropy using the symmetry of graphical representations. In addition, a reverse causation analysis was carried out to exclude the possibility that T2D causally affected tea intake using T2D-associated SNPs as IVs.

### Power Analyses

It is well known that a lack of statistical power may lead to unobserved true causal effects. We carried out the mRnd (http://cnsgenomics.com/shiny/mRnd/) method for power analysis of the MR to identify a nonzero causal effect of tea intake on T2D (Brion et al., 2013).

### Statistical Software

We performed MR analyses using R packages “MendelianRandomization” (Yavorska and Burgess, 2017), “MRPRESSO” (Verbanck et al., 2018), and “mr.raps” (Zhao et al., 2020). Statistical analyses were conducted in the R (version 4.0.0) environment. The statistical significance level was set at *p* < 0.05 throughout our study.

## Results

### Selection of Instrumental Variables

A total of 24 SNPs for tea intake were obtained using rigorous procedures (Figure 2), and relevant information was extracted from summary statistics of the same SNPs from T2D GWAS and harmonized, giving 24 index SNPs as valid IVs, which explained about 0.29% of the phenotypic variations in tea intake (Supplementary Table S2). rs9624470 and rs1057868 showed strong associations with tea intake (*p* = 1.32 × 10^−29^, *p* = 3.09 × 10^−23^, respectively). The overall *F* statistic was 44.64, and the *F* statistic for each IV was above 10. Therefore, we could conclude that weak instrument bias would not substantially influence causal inference in our MR analysis.

### Causal Effect in the Main Analysis

**FIGURE 3:**
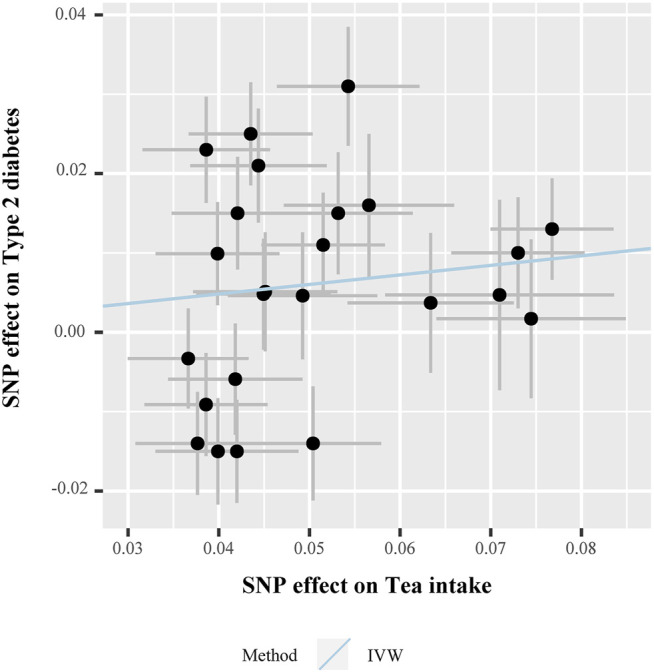
Scatter plot for the SNP effect size estimate. Relationship between the SNP effect size estimate of tea intake (*x*-axis) and the corresponding effect size estimate of T2D (*y*-axis). Each black dot represents a valid instrumental SNP. A total of 24 SNPs were analyzed. The horizontal and vertical short lines through the dots represent the 95% CIs of the SNP effect on tea intake and T2D effect, respectively. The slope of fitted lines (blue line) represents the estimated causal effect of tea intake on T2D using the IVW method. Abbreviations: SNPs, single-nucleotide polymorphisms; T2D, type 2 diabetes; IVW, inverse variance-weighted method.

In the MR analysis, the odds ratio (OR) per unit SD change of tea intake (SD: 2.85 cups/day) for T2D, HbA1c, FPG, FSI, and HOMA-IR levels, obtained using the IVW method, was as follows: 0.949 (95% CI 0.844–1.067, *p* = 0.383), 0.994 (95% CI 0.975–1.013, *p* = 0.554), 0.996 (95% CI 0.978–1.015, *p* = 0.703), 0.968 (95% CI 0.948–0.986, *p* = 0.001), and 0.953 (95% CI 0.900–1.009, *p* = 0.102), respectively. Figure 3 presents the relationship of SNP effect size between tea intake and T2D for each IV.

### Sensitivity Analyses

**FIGURE 4:**
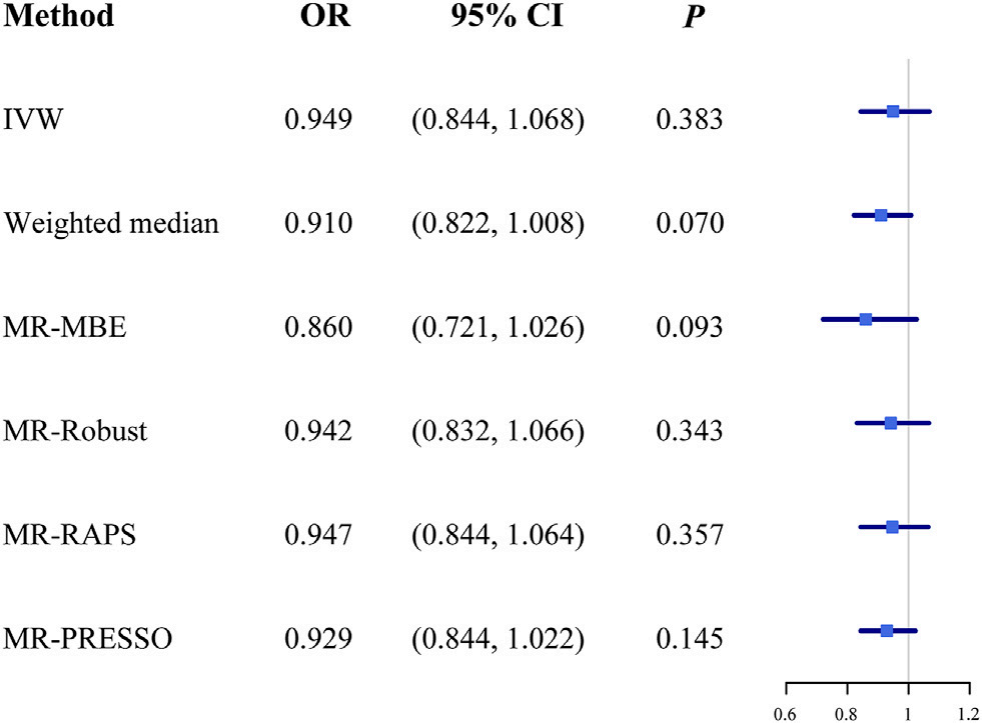
The causal effect estimates from various MR methods. The odds ratios of tea intake on T2D are displayed as a black solid box. The 95% CIs are shown as horizontal gray lines. Abbreviations: MR, Mendelian randomization; MR-MBE, MR Mode-based estimate; MR-Robust, inverse variance-weighted method using robust regression; MR-RAPS, Robust Adjusted Profile Score; MR-PRESSO, Mendelian Randomization Pleiotropy RESidual Sum and Outlier; OR, odds ratio.

**FIGURE 5:**
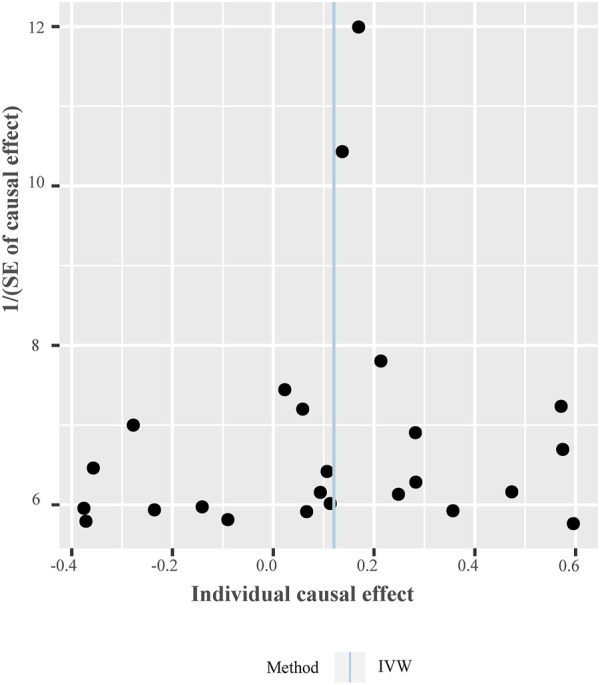
Funnel plot of individual causal effect between tea intake and T2D. Causal effect for each instrumental variable is displayed by the black dots to assess potential asymmetry through the funnel plot. Each black dot represents a valid instrumental SNP. The vertical blue line represents the estimated causal effect using all instrumental SNPs from IVW methods. Abbreviations: IVW, inverse variance-weighted method; SNPs, single-nucleotide polymorphisms.

**FIGURE 6:**
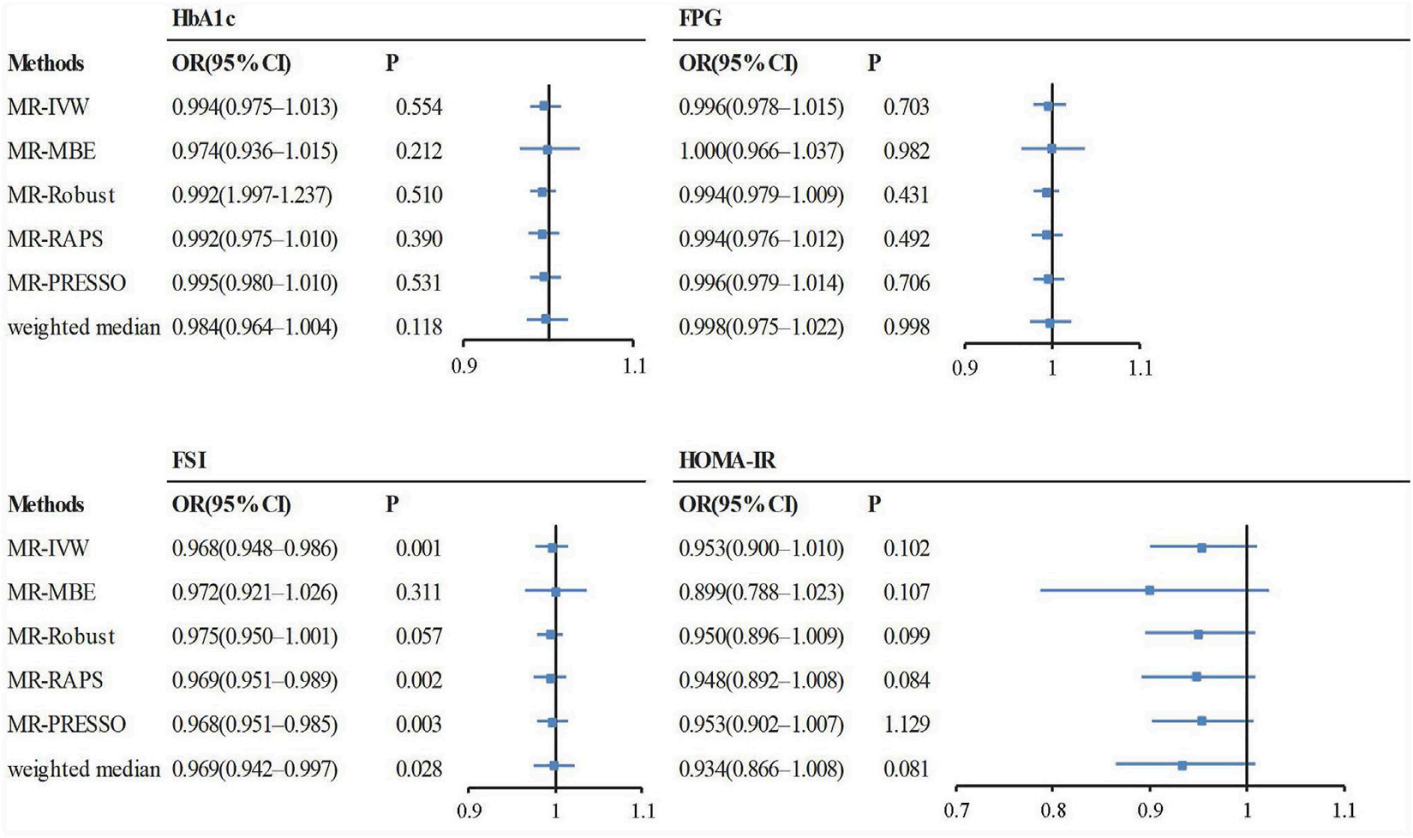
The causal effect estimates from various MR methods. The odds ratios of tea intake on glycemic traits are displayed as a black solid box. The 95% CIs are shown as horizontal gray lines. Abbreviations: FPG, fasting plasma glucose; HbA1c, hemoglobin A1c; FSI, fasting serum insulin; HOMA-IR, homeostasis model of insulin resistance; MR, Mendelian randomization; MR-MBE, MR Mode-based estimate; MR-Robust, inverse variance-weighted method using robust regression; MR-RAPS, Robust Adjusted Profile Score; MR-PRESSO, Mendelian Randomization Pleiotropy RESidual Sum and Outlier; OR, odds ratio.

The OR estimates between tea intake and T2D were 0.910 (95% CI 0.822–1.008; *p* = 0.070) for the weighted median method, 0.860 (95% CI 0.721–1.026; *p* = 0.093) for MR-MBE, 0.942 (95% CI 0.832–1.066; *p* = 0.343) for MR-Robust, and 0.947 (95% CI 0.844–1.064; *p* = 0.357) for MR-RAPS (Figure 4). The MR-Egger regression is similar to the null estimate (OR = 0.996, 95% CI 0.602–1.648, *p* = 0.656), with an intercept of 0.003 (95% CI −0.022–0.028, *p* = 0.803), suggesting that there is no directional pleiotropy in our two-sample MR. A funnel plot for individual causal effect estimates is shown in Figure 5. The diagnostic funnel plot shows a visually apparent symmetry, which excluded the possible influence of directional pleiotropy on our estimates. LOO analysis further showed that no single instrumental SNP could substantially influence the causal effect estimation (Supplementary Table S3). MR-PRESSO identified three outliers that might violate the causal effect estimate. After removal of these pleiotropic SNPs, the OR estimate was 0.929 (95% CI 0.844–1.022; *p* = 0.145), indicating no causal association between tea intake and T2D. Figure 6 presents the relationship between tea intake and glycemic traits using sensitivity analyses. In the bidirectional analyses, the T2D–tea intake causal model was not significant (MR-IVW, OR = 0.983; 95% CI 0.959–1.007; *p* = 0.169), indicating no reverse causation.

### Power Analyses

**FIGURE 7:**
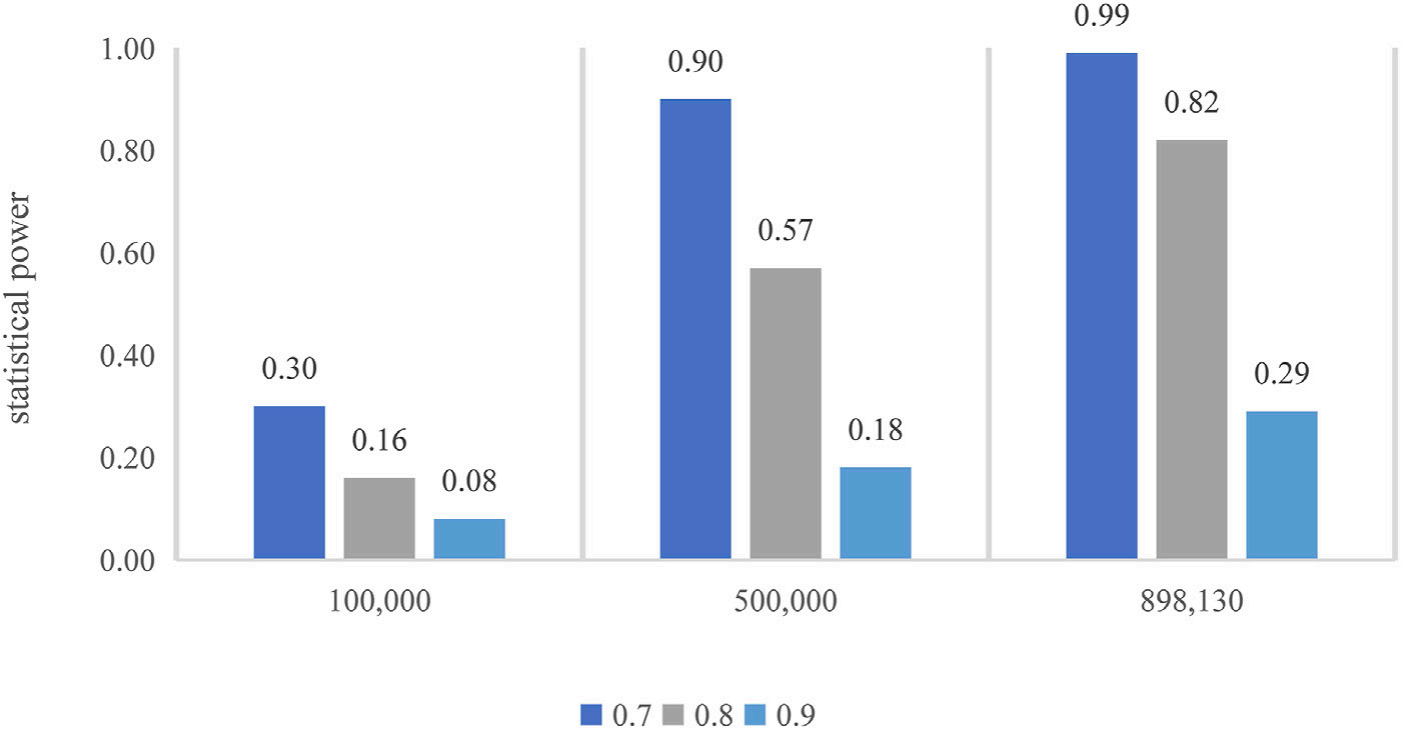
Power calculation for the MR. The top of each bar represents the estimated power, with the *x*-axis indicating the different sample sizes.

In the mRnd method, we set the phenotypic variance explained (PVE) to 0.29%, which is equal to the total phenotypic variance of tea intake explained by all the valid IVs. To examine whether our MR analyses have sufficient statistical power to detect causal effects, as noted in observational studies, we additionally conducted a power analysis by assuming several different ORs (i.e., 0.70, 0.80, or 0.90) in the power analysis. Note that the determination of these ORs was based on effect estimates between tea intake and T2D from previous cohort studies. Specifically, in a dose–response meta-analysis of cohort studies on tea consumption and risk of T2D (Yang et al., 2014a), an increase of two cups/day of tea consumption was associated with a 4.60% (95% CI, 0.90–8.10%) reduced risk of T2D. An earlier meta-analysis revealed that tea consumption of four cups/day (relative risk, 0.80; 95% CI, 0.70–0.93) might play a role in the prevention of T2D (Imamura et al., 2019). In addition, the significance level α was set to 0.05, and the proportion of T2D cases was 8.25%, which was calculated from the T2D GWAS. We also calculated the power for different sample sizes (i.e., 100,000, 500,000, and 898,130). The results implied that our MR analysis would have moderate-to-high power (Figure 7). For example, with the current sample size (898,130), the estimated statistical power was 29%, 82%, and 99% when the OR was 0.90, 0.80, and 0.70, respectively.

## Discussion

The findings of our MR analyses suggested that tea intake does not influence the risk of T2D and several crucial glycemic traits including HbA1c, FPG, and HOMA-IR levels. The study took great advantage of genetic instruments in the MR study and benefited from the large sample size of the GWAS. The results were verified through various sensitivity analyses using multiple MR methods with different model assumptions, followed by a comprehensive evaluation of the influence of pleiotropy effects and outliers. All the analyses suggested that the findings were consistent and robust. In addition, bidirectional analyses indicated no reverse causation.

Our findings were consistent with some previous epidemiological studies, which investigated the association between tea intake and T2D, including cross-sectional studies (Yamaji et al., 2004), cohort studies (Oba et al., 2010; Imamura et al., 2019), and meta-analyses (Isogawa et al., 2003). More importantly, our results were also consistent with some meta-analyses of RCTs, except for the glycemic trait FSI. A meta-analysis of seven RCTs showed that the consumption of green tea did not lower the levels of FPG, FSI, and 2-h plasma glucose in the oral glucose tolerance test, HbA1c, and HOMA-IR in populations at risk of T2D (Wang et al., 2014). A meta-analysis of 10 RCTs illustrated that tea or tea extracts did not decrease the levels of FPG (Li et al., 2016). Another meta-analysis of 10 RCTs also concluded that in patients with T2D, the levels of HbA1c, FPG, and HOMA-IR in the green tea or green tea extract treatment group did not decrease, as compared with the placebo group (Yu et al., 2017). In addition, a recently published meta-analysis of RCTs also indicated that supplementary green tea drinking had no significant effect on FPG, fasting insulin, HbA1c, and HOMA-IR in patients with T2D (Asbaghi et al., 2021). Note that tea intake had a negative effect on FSI levels but had no effect on FPG and HbA1c levels in our study. However, the diagnosis of T2D was mainly based on FPG and HbA1c, which shows that our results are not contradictory.

To the best of our knowledge, there are a total of six meta-analyses of RCTs with human participants, which explore the effects of tea or tea extracts on FPG or HbA1c (Liu et al., 2013; Zheng et al., 2013; Wang et al., 2014; Li et al., 2016; Yu et al., 2017; Asbaghi et al., 2021), and two of them were inconsistent with our findings (Liu et al., 2013; Zheng et al., 2013). This might be because the original population in these two studies included both healthy people and patients with chronic diseases other than T2D, such as metabolic syndrome, obesity, and hypertension, leading to confounding findings on the effect of tea intake on the risk of T2D. Although some animal studies indicated that certain components of tea lowered FPG and improved insulin resistance (Nishiumi et al., 2010; Li et al., 2020b), we cannot rule out the possibility of differences in glucometabolism across species. In addition, the composition of tea is very complex, and different components may affect insulin sensitivity and glycemic control in different ways (Alipour et al., 2018). For example, more studies have focused on the relationship between tea polyphenols and caffeine in tea and glucose metabolism. Epigallocatechin gallate is an important tea polyphenol, which can improve insulin resistance by activating the 5′-adenylic acid mitogen-activated protein kinase pathway (Lin and Lin, 2008) or upregulating the level of insulin signaling protein (Qin et al., 2010). Polyphenolic compounds may affect glucose metabolism by inhibiting the absorption of glucose in the intestines and stimulating insulin secretion (Hanhineva et al., 2010). However, caffeine may impair insulin sensitivity by increasing plasma epinephrine and free fatty acid (FFA) levels (Greer et al., 20012001; Keijzers et al., 2002). The relationship between various components of tea and glucose metabolism requires further investigation.

This study had several strengths. To the best of our knowledge, it is the first attempt to investigate the genetic causal relationship between tea intake and T2D through a two-sample MR framework using GWAS summary statistics, adding to existing evidence about the prevention of T2D. Second, the results were consistent through various sensitivity analyses using different MR methods with different model assumptions, suggesting that the findings were robust and convincing. Third, we used funnel plots, MR-Egger, and MR-PRESSO to test the potential horizontal pleiotropy and outliers. However, this study had some limitations. First, the DIAGRAM consortium includes association summary statistics for T2D from the UK Biobank (Yamaji et al., 2004). The sample overlap of the two datasets could bias the estimated causal effect. However, our strong IVs (i.e., *F* statistic greater than 10) could reduce bias from sample overlap. In our study, the overall *F* statistic was 44.64, and the *F* statistic for each IV was above 10. Second, we emphasize that the power in our analysis is relatively limited in observing a weak effect of tea intake of T2D, which may partly be due to the low proportion of the variance in tea intake explained by the valid instrumental SNPs. Third, it should be noted that our study was primarily based on available GWAS summary data. However, there is no GWAS on distinct types of tea, leading to the difficulty in deducing different effects of the types of tea on the causal association between tea intake and T2D. Fourth, MR may be susceptible to collider bias due to the results of genome-wide data adjusted for covariates (Davies et al., 2018). For binary outcomes, there is also potential bias due to the non-collapsibility of the OR (Sheehan and Didelez, 2020). Fifth, it should be noted that our study was primarily based on available summary data, making it difficult to deduce the direct effect of gender on the causal association between tea intake and T2D. Finally, the results from MR reflect the effect of drinking tea on T2D throughout the lifetime, so the short-term effect of drinking tea on T2D needs additional investigation.

## Conclusion

To conclude, our MR study did not provide genetic evidence of the causal effect of tea intake on T2D and several glycemic traits including HbA1c, FPG, and HOMA-IR levels. Our results implied that the evidence for tea drinking as a preventative measure for T2D is still insufficient and that some of the previous findings on the association between tea consumption and T2D or glycemic traits may be biased by confounders. More experimental studies are required for further validation.

## Data Availability

Publicly available datasets were analyzed in this study. This data can be found here: https://atlas.ctglab.nl/traitDB/3255, http://diagram-consortium.org/.
