# Supplementary material for: A Mendelian Randomization Study of the Effect of Tea Intake on Type 2 Diabetes
Source: Front Genet. 2022 Mar 29;13:835917. doi: 10.3389/fgene.2022.835917 (PMC9001914; doi:10.3389/fgene.2022.835917)
Supplement: Supplementary file 1 [file DataSheet1.docx]

Supplementary Material

# Supplementary Tables

**Table S1:** The comparison and complement of different methods**.**

| **Category** | **Methods** | **Strengths and/or weaknesses** |
| --- | --- | --- |
| Consensus methods | the weighted median method | Relax the instrumental variable assumptions, robust to outliers, may be less efficient |
|  | MR Mode-based estimate | Relax the instrumental variable assumptions, robust to outliers |
| Modelling methods | MR Robust Adjusted Profile Score | Both systematic and idiosyncratic pleiotropy and performed excellently |
|  | MR Egger regression | Evaluate the directional pleiotropy of IVs according to its intercept |
|  | inverse-variance weighted method using robust regression | Efficient with valid IVs, high false-positive rate with several invalid IVs |
| Outlier-robust methods | Mendelian Randomisation Pleiotropy RESidual Sum and Outlier | Evaluate the bias from outliers and assess the heterogeneity of IVs |
|  | leave-one-out cross-validation analysis | Examine whether there are outliers and whether these outliers affect results |

Abbreviations: MR, Mendelian randomization; IV, instrumental variable.

**Table S2: Index instrumental SNPs for tea intake and the effects, standard errors for T2D.**

| SNP | Chr | Position (GRCh37) | Allele1 | Allele2 | tea intake | | | | | |  | T2D | | |
| --- | --- | --- | --- | --- | --- | --- | --- | --- | --- | --- | --- | --- | --- | --- |
|  |  |  |  |  | Beta | SE | N | P | PVE | F |  | Beta | SE | P |
| rs1057868 | 7 | 75615006 | T | C | 0.0730 | 0.0074 | 373481 | 3.09E-23 | 2.64E-04 | 98.6169 |  | 0.0198 | 0.0127 | 0.1213 |
| rs10741694 | 11 | 16286183 | T | C | -0.0399 | 0.0069 | 369858 | 7.24E-09 | 9.05E-05 | 33.4745 |  | 0.0227 | 0.0119 | 0.0519 |
| rs10845296 | 12 | 11184140 | G | A | -0.0493 | 0.0082 | 373481 | 2.28E-09 | 9.56E-05 | 35.7236 |  | 0.0334 | 0.0142 | 0.0172 |
| rs11022752 | 11 | 13307622 | G | A | 0.0444 | 0.0075 | 369978 | 3.82E-09 | 9.38E-05 | 34.7205 |  | -0.0232 | 0.0131 | 0.0772 |
| rs11768350 | 7 | 17561651 | C | T | -0.0634 | 0.0092 | 369740 | 4.90E-12 | 1.29E-04 | 47.7246 |  | 0.0198 | 0.0158 | 0.2020 |
| rs12302023 | 12 | 72715420 | C | A | -0.0421 | 0.0073 | 371733 | 7.43E-09 | 8.99E-05 | 33.4224 |  | 0.0266 | 0.0126 | 0.0319 |
| rs12591786 | 15 | 60902512 | T | C | -0.0566 | 0.0094 | 361521 | 1.73E-09 | 1.00E-04 | 36.2506 |  | -0.0209 | 0.0163 | 0.2010 |
| rs13282783 | 8 | 22088975 | T | C | -0.0449 | 0.0074 | 367111 | 1.43E-09 | 9.98E-05 | 36.6363 |  | -0.0164 | 0.0129 | 0.2015 |
| rs1640299 | 22 | 20098359 | G | T | -0.0366 | 0.0067 | 371598 | 3.89E-08 | 8.13E-05 | 30.2045 |  | -0.0192 | 0.0115 | 0.0962 |
| rs2279844 | 17 | 40819809 | A | G | -0.0435 | 0.0068 | 373063 | 2.06E-10 | 1.08E-04 | 40.4230 |  | -0.0161 | 0.0119 | 0.1744 |
| rs2315025 | 19 | 19426609 | T | C | 0.0386 | 0.0070 | 372339 | 4.15E-08 | 8.07E-05 | 30.0682 |  | 0.0286 | 0.0122 | 0.0197 |
| rs3826792 | 19 | 47776698 | C | T | -0.0377 | 0.0069 | 359819 | 4.15E-08 | 8.36E-05 | 30.0850 |  | -0.0088 | 0.0119 | 0.4553 |
| rs389750 | 16 | 63022689 | A | G | 0.0451 | 0.0080 | 372438 | 1.46E-08 | 8.62E-05 | 32.1057 |  | 0.0227 | 0.0137 | 0.0929 |
| rs4721671 | 7 | 17978520 | C | G | -0.0418 | 0.0074 | 363409 | 1.73E-08 | 8.74E-05 | 31.7742 |  | 0.0109 | 0.0128 | 0.3991 |
| rs4817505 | 21 | 34343828 | C | T | 0.0399 | 0.0068 | 370121 | 5.33E-09 | 9.20E-05 | 34.0589 |  | -0.0232 | 0.0119 | 0.0503 |
| rs61826916 | 1 | 174585186 | C | T | -0.0515 | 0.0068 | 373058 | 4.12E-14 | 1.53E-04 | 57.1280 |  | 0.0129 | 0.0118 | 0.2590 |
| rs72799105 | 5 | 152048633 | G | A | -0.0504 | 0.0075 | 370744 | 2.14E-11 | 1.21E-04 | 44.8467 |  | -0.0298 | 0.0131 | 0.0227 |
| rs73147887 | 20 | 62890294 | G | C | 0.0532 | 0.0082 | 357425 | 1.02E-10 | 1.17E-04 | 41.7887 |  | 0.0050 | 0.0143 | 0.7204 |
| rs73389047 | 12 | 107320452 | G | A | -0.0710 | 0.0126 | 372431 | 1.91E-08 | 8.48E-05 | 31.5748 |  | 0.0276 | 0.0217 | 0.2095 |
| rs73424602 | 22 | 41461176 | T | C | -0.0420 | 0.0068 | 370804 | 6.16E-10 | 1.03E-04 | 38.2723 |  | 0.0080 | 0.0117 | 0.4987 |
| rs74904971 | 4 | 89050026 | A | C | -0.0745 | 0.0105 | 373430 | 1.07E-12 | 1.36E-04 | 50.7568 |  | 0.0000 | 0.0181 | 0.9802 |
| rs7529194 | 1 | 150622620 | A | T | 0.0386 | 0.0068 | 373470 | 1.38E-08 | 8.63E-05 | 32.2244 |  | 0.0050 | 0.0118 | 0.6811 |
| rs9624470 | 22 | 24820268 | G | A | -0.0768 | 0.0068 | 366477 | 1.32E-29 | 3.48E-04 | 127.6863 |  | 0.0080 | 0.0118 | 0.5140 |
| rs994270 | 6 | 51187787 | G | C | 0.0543 | 0.0079 | 371007 | 5.24E-12 | 1.28E-04 | 47.6003 |  | -0.0246 | 0.0137 | 0.0733 |

Abbreviations: SNP, single nucleotide polymorphism; Chr, chromosome; A1, effect allele; A2 other allele; Beta, SNP effect size; SE, standard error; PVE, proportion of variance in phenotype explained by a given SNP; N, sample size; T2D, type 2 diabetes.

**Table S3: Estimated causal effects and 95%CIs for tea intake on T2D in the leave-one-out analysis by removing one instruments each time using the IVW method.**

| SNP | BETA | SE | 95% CI | P | I2 |
| --- | --- | --- | --- | --- | --- |
| rs61826916 | -0.0431 | 0.0620 | (-0.1647,0.0785) | 0.4872 | 76.4330 |
| rs11022752 | -0.0378 | 0.0596 | (-0.1547,0.0791) | 0.5261 | 75.0172 |
| rs10741694 | -0.0418 | 0.0606 | (-0.1606,0.0770) | 0.4901 | 75.8745 |
| rs73389047 | -0.0516 | 0.0619 | (-0.1728,0.0697) | 0.4044 | 76.8472 |
| rs10845296 | -0.0506 | 0.0619 | (-0.1720,0.0707) | 0.4136 | 76.8326 |
| rs12302023 | -0.0425 | 0.0608 | (-0.1616,0.0765) | 0.4838 | 76.0010 |
| rs12591786 | -0.0638 | 0.0605 | (-0.1823,0.0547) | 0.2916 | 75.6753 |
| rs389750 | -0.0573 | 0.0616 | (-0.1780,0.0633) | 0.3519 | 76.5984 |
| rs2279844 | -0.0771 | 0.0558 | (-0.1865,0.0323) | 0.1672 | 71.3282 |
| rs2315025 | -0.0710 | 0.0570 | (-0.1827,0.0406) | 0.2123 | 72.7338 |
| rs3826792 | -0.0646 | 0.0598 | (-0.1817,0.0525) | 0.2798 | 75.2311 |
| rs73147887 | -0.0422 | 0.0613 | (-0.1624,0.0780) | 0.4917 | 76.1893 |
| rs4817505 | -0.0455 | 0.0614 | (-0.1659,0.0750) | 0.4594 | 76.4780 |
| rs1640299 | -0.0563 | 0.0616 | (-0.1770,0.0645) | 0.361 | 76.6764 |
| rs9624470 | -0.0355 | 0.0643 | (-0.1615,0.0906) | 0.5813 | 76.2849 |
| rs73424602 | -0.0407 | 0.0607 | (-0.1596,0.0783) | 0.5031 | 75.8231 |
| rs74904971 | -0.0558 | 0.0623 | (-0.1779,0.0664) | 0.3710 | 76.7692 |
| rs72799105 | -0.0665 | 0.0604 | (-0.1849,0.0519) | 0.2708 | 75.4093 |
| rs994270 | -0.0276 | 0.0573 | (-0.1398,0.0847) | 0.6305 | 72.5850 |
| rs11768350 | -0.0517 | 0.0623 | (-0.1739,0.0704) | 0.4067 | 76.8484 |
| rs4721671 | -0.0492 | 0.0618 | (-0.1703,0.0719) | 0.4259 | 76.7775 |
| rs1057868 | -0.0715 | 0.0625 | (-0.1940,0.0510) | 0.2528 | 75.7556 |
| rs13282783 | -0.0578 | 0.0617 | (-0.1787,0.0630) | 0.3483 | 76.5834 |

I^2^: a statistic which quantifies the effect of heterogeneity, as the total variation due to heterogeneity across causal estimates amongst all SNPs. Larger I^2^ indicates greater heterogeneity, with cut offs for the low, moderate, and high heterogeneity being 25%, 50% and 75%, respectively. Abbreviations: SNP, single nucleotide polymorphism; SE, standard error; CI, confidence interval.

# Supplementary Figures

**
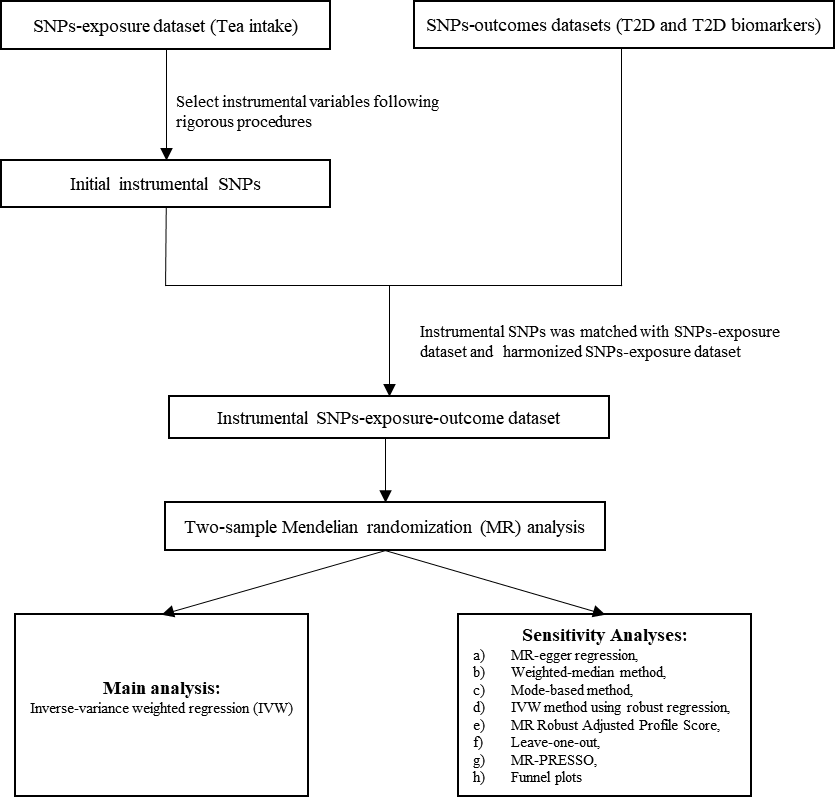
**

**Figure S1: Study design with a flow diagram.**
